# Supplementary material for: Culturally adapted quasi-experimental interventions for reducing entomophobia and disgust: A study among older adults in Iran and Malaysia
Source: Glob Ment Health (Camb). 2026 Apr 16;13:e101. doi: 10.1017/gmh.2026.10167 (PMC13231228; doi:10.1017/gmh.2026.10167)
Supplement: Soltani et al. supplementary material [file S2054425126101678sup001.docx]

**SUPPLEMENTAL FILE 1. DETAILED BASELINE SCORES**

**Descriptive Characteristics of Participants:**

All Samples

The demographic characteristics of the older adults in this study revealed a sample size of N = 151, with the majority being Iranian (N = 82, 54.3%) and the remainder Malaysian (N = 69, 45.7%). Gender distribution showed a significant female predominance, with females constituting N = 104 (68.9%) compared to males N = 47 (31.1%). Ethnicity was predominantly Persian (N = 82, 54.3%), followed by Malay (N = 30, 19.9%), Indian (N = 19, 12.6%), and Chinese (N = 20, 13.2%). In terms of age classification, most participants fell within the 60-75 years range (N = 139, 92.1%), while fewer were in the 75-90 years range (N = 12, 7.9%). Chronic conditions were prevalent, with N = 113 (74.8%) reporting at least one chronic condition. Common chronic conditions included high blood pressure (N = 17, 11.3%), heart disease (N = 17, 11.3%), and depression (N = 25, 16.6%). The duration of chronic conditions varied, with 29.1% (N = 44) having them for 2-5 years. Sleeping patterns indicated that the majority slept between 5 and 8 hours per night (N = 89, 58.9%), while fewer slept less than 5 hours (N = 24, 15.9%) or more than 8 hours (N = 38, 25.2%). General health perceptions were largely positive, with many participants describing themselves as "Very healthy – I feel good!" (N = 57, 37.7%) or "Healthy – I have a few problems that are well-managed" (N = 63, 41.7%). A smaller proportion reported poorer health, such as "To some extent – I have good days and bad days" (N = 19, 12.6%) or "Poor – My health significantly limits what I can do" (N = 12, 7.9%). Living arrangements showed that most participants lived with others (N = 114, 75.5%), while a smaller percentage lived alone (N = 37, 24.5%). Family relationships were diverse, with many living with spouses (N = 47, 31.1%) or children (N = 27, 17.9%). Marital status indicated that most were married (N = 77, 51.0%), followed by widowed (N = 38, 25.2%) and divorced (N = 14, 9.3%). Fear of insects was a key focus, with scorpions being the most feared insect (N = 58, 38.4%), followed by spiders (N = 28, 18.5%). Large flying insects were the biggest fear for many (N = 78, 51.7%), followed by small flying insects (N = 55, 36.4%). Reasons for fear included bites (N = 35, 23.2%), being pathogenic (N = 7, 4.6%), and disgust (N = 10, 6.6%). Knowledge about insect pathogenicity was widespread, with N = 100 (66.2%) aware of it. Additionally, N = 97 (64.2%) reported looking for safe places when encountering insects.

Nationality

Iranian and Malaysian older adults exhibited notable differences in demographics and attitudes toward insects. Iranians constituted the majority of the sample (N = 82, 54.3%), while Malaysians made up N = 69 (45.7%). Gender distribution showed that females were predominant in both nationalities, but slightly more so among Malaysians (N = 44, 29.1%) compared to Iranians (N = 60, 39.7%). Ethnicity was a distinguishing factor, as all Iranian participants identified as Persian (N = 82, 54.3%), whereas Malaysians belonged to various ethnic groups such as Malay (N = 30, 19.9%), Indian (N = 19, 12.6%), and Chinese (N = 20, 13.2%). Chronic conditions were more prevalent among Iranians (N = 66, 43.7%) compared to Malaysians (N = 47, 31.1%). Depression was significantly higher among Iranians (N = 18, 11.9%) compared to Malaysians (N = 7, 4.6%), while respiratory diseases were more common among Malaysians (N = 9, 6.0%) than Iranians (N = 7, 4.6%). Sleeping patterns also differed, with Iranians more likely to sleep more than 8 hours (N = 19, 12.6%) compared to Malaysians (N = 19, 12.6%). Fear-related responses showed that scorpions were more feared by Iranian participants (N = 37, 24.5%) compared to Malaysians (N = 21, 13.9%). Large flying insects were the biggest fear for both groups, but slightly more so among Iranians (N = 42, 27.8%) than Malaysians (N = 27, 17.9%). Knowledge about insect pathogenicity was higher among Iranians (N = 57, 37.7%) compared to Malaysians (N = 43, 28.5%). Behavioral responses, such as looking for safe places, were also more common among Iranians (N = 51, 33.8%) than Malaysians (N = 46, 30.5%), suggesting differing levels of fear and coping mechanisms.

Gender

Gender-based differences among older adults highlighted significant variations in demographics and attitudes toward insects. Females made up the majority of the sample (N = 104, 68.9%), with males constituting N = 47 (31.1%). Ethnic diversity was more pronounced among females, with 39.7% identifying as Persian (N = 60), compared to 14.6% of males (N = 22). Age classification showed that females were more likely to fall within the 60-75 age range (N = 93, 61.6%) compared to males (N = 46, 30.5%), while males were slightly more likely to be in the 75-90 age range (N = 1, 0.7%). Chronic conditions were more prevalent among females (N = 77, 51.0%) compared to males (N = 36, 23.8%). Depression was particularly higher among females (N = 19, 12.6%) than males (N = 6, 4.0%), reflecting potential gender-specific health concerns. Sleep patterns indicated that females were more likely to sleep more than 8 hours (N = 28, 18.5%) compared to males (N = 10, 6.6%), while males were more likely to sleep less than 5 hours (N = 6, 4.0%). Fear of insects was more pronounced among females, with scorpions being the most feared insect (N = 39, 25.8% of females vs. N = 19, 12.6% of males). Large flying insects were the biggest fear for females (N = 56, 37.1%) compared to males (N = 22, 14.6%). Reasons for fear included bites (N = 27, 17.9% of females vs. N = 8, 5.3% of males) and disgust (N = 6, 4.0% of males vs. N = 10, 6.6% of females). Females were also more likely to look for safe places when encountering insects (N = 64, 42.4%) compared to males (N = 33, 21.9%), indicating a stronger behavioral response to fear. Overall, these findings suggest that gender plays a critical role in shaping fear-related attitudes and behaviors.

**Comprehensive Descriptive Statistical Report**

This report integrates data from all three sheets (Sheet1, Sheet2, and Sheet3) to provide a detailed analysis of various continuous variables among older adults in Iranian and Malaysian populations. The dataset includes demographic information, insect phobia measurements using the Entomophobia Visual Instrument (EVI) , and the OAEAS questionnaire scores, segmented by nationality, gender, pet keeping, knowledge about insects' pathogenicity, teaching models, chronic conditions, looking for safe place behavior, and other factors.

Introduction to Measurement Tools:

1- Entomophobia Visual Instrument (EVI): This tool uses images of 10 insects (locusts, beetles, butterflies, dragonflies, spiders, bees, ants, millipedes, ticks, flies) to assess the severity of insect phobias. Participants rate their fear and disgust levels on a scale of 0 (none) to 2 (highest). Scores range from 0 to 20, with higher scores indicating greater fear or disgust.

2- OAEAS Questionnaire: A paper-based questionnaire with 20 Likert-scale items (5-point options) measures insect phobia. Scores range from 20 (lowest) to 100 (highest), where higher scores indicate more severe insect phobia.

Years Old

The overall mean age of the sample was Mean = 68.37 years (SD = 6.13, IQR = 8). When broken down by nationality, Iranian older adults had a mean age of Mean = 67.52 years (SD = 5.89, IQR = 8.75), while Malaysian older adults had a mean age of Mean = 69.38 years (SD = 6.31, IQR = 9). By gender, male participants had a mean age of Mean = 67.96 years (SD = 5.42, IQR = 6), and female participants had a mean age of Mean = 68.56 years (SD = 6.45, IQR = 9.25).

For ethnic groups, Persian older adults had a mean age of Mean = 67.52 years (SD = 5.89, IQR = 8.75), Malay older adults had Mean = 68.87 years (SD = 5.07, IQR = 6), Indian older adults had Mean = 69.11 years (SD = 6.73, IQR = 11.5), and Chinese older adults had Mean = 70.40 years (SD = 7.67, IQR = 11).

Older adults who did not keep pets had a mean age of Mean = 68.39 years (SD = 5.69, IQR = 8), while pet owners had a mean age of Mean = 68.33 years (SD = 6.96, IQR = 10.25). Those without knowledge about insects' pathogenicity had a mean age of Mean = 69.82 years (SD = 6.59, IQR = 9.5), while those with knowledge had a mean age of Mean = 67.63 years (SD = 5.78, IQR = 7.25).

Older adults who looked for safe places had a mean age of Mean = 67.64 years (SD = 5.45, IQR = 6), while those who did not had a mean age of Mean = 69.69 years (SD = 7.07, IQR = 11.75). Participants with chronic conditions had a mean age of Mean = 68.05 years (SD = 5.73, IQR = 8), while those without had a mean age of Mean = 69.32 years (SD = 7.20, IQR = 11).

EVI: Disgusting Pre

The overall mean score for EVI: Disgusting Pre was Mean = 7.72 (SD = 2.74, IQR = 4). Iranian older adults scored Mean = 7.46 (SD = 2.88, IQR = 5), while Malaysian older adults scored Mean = 8.03 (SD = 2.56, IQR = 4). Male participants scored higher with Mean = 8.23 (SD = 2.17, IQR = 3), compared to females who scored Mean = 7.49 (SD = 2.95, IQR = 5).

By ethnicity, Persian older adults scored Mean = 7.46 (SD = 2.88, IQR = 5), Malay older adults scored Mean = 7.73 (SD = 2.70, IQR = 2), Indian older adults scored Mean = 7.58 (SD = 2.48, IQR = 5), and Chinese older adults scored Mean = 8.90 (SD = 2.31, IQR = 4).

For pet keeping, non-pet owners scored Mean = 8.07 (SD = 2.35, IQR = 3), while pet owners scored Mean = 7.06 (SD = 3.29, IQR = 6). Older adults without knowledge about insects' pathogenicity scored Mean = 7.25 (SD = 2.95, IQR = 5), while those with knowledge scored Mean = 7.96 (SD = 2.62, IQR = 3).

Older adults who looked for safe places scored Mean = 7.91 (SD = 2.67, IQR = 4), while those who did not scored Mean = 7.39 (SD = 2.87, IQR = 5). Participants with chronic conditions scored Mean = 7.92 (SD = 2.51, IQR = 3), while those without scored Mean = 7.13 (SD = 3.31, IQR = 6).

EVI: Phobia Pre

The overall mean score for EVI: Phobia Pre was Mean = 7.23 (SD = 2.85, IQR = 5). Iranian older adults scored Mean = 7.49 (SD = 2.65, IQR = 4.75), while Malaysian older adults scored Mean = 6.91 (SD = 3.07, IQR = 4). Male participants scored Mean = 7.53 (SD = 2.78, IQR = 5), and female participants scored Mean = 7.09 (SD = 2.89, IQR = 4.25).

By ethnicity, Persian older adults scored Mean = 7.49 (SD = 2.65, IQR = 4.75), Malay older adults scored Mean = 6.60 (SD = 3.29, IQR = 5), Indian older adults scored Mean = 6.42 (SD = 2.89, IQR = 4), and Chinese older adults scored Mean = 7.85 (SD = 2.83, IQR = 4.25).

For pet keeping, non-pet owners scored Mean = 7.42 (SD = 2.71, IQR = 5), while pet owners scored Mean = 6.85 (SD = 3.10, IQR = 5.25). Older adults without knowledge about insects' pathogenicity scored Mean = 6.98 (SD = 2.88, IQR = 4), while those with knowledge scored Mean = 7.35 (SD = 2.85, IQR = 5).

Older adults who looked for safe places scored Mean = 7.33 (SD = 2.90, IQR = 5), while those who did not scored Mean = 7.04 (SD = 2.79, IQR = 4). Participants with chronic conditions scored Mean = 7.29 (SD = 2.83, IQR = 5), while those without scored Mean = 7.03 (SD = 2.95, IQR = 5.75).

EVI: Disgusting Post

The overall mean score for EVI: Disgusting Post was Mean = 6.91 (SD = 2.19, IQR = 3). Iranian older adults scored Mean = 6.83 (SD = 2.45, IQR = 3), while Malaysian older adults scored Mean = 7.00 (SD = 1.86, IQR = 2). Male participants scored Mean = 7.11 (SD = 1.72, IQR = 2), and female participants scored Mean = 6.82 (SD = 2.38, IQR = 3.25).

By ethnicity, Persian older adults scored Mean = 6.83 (SD = 2.45, IQR = 3), Malay older adults scored Mean = 6.97 (SD = 2.04, IQR = 1.75), Indian older adults scored Mean = 6.79 (SD = 1.58, IQR = 2), and Chinese older adults scored Mean = 7.25 (SD = 1.89, IQR = 3).

For pet keeping, non-pet owners scored Mean = 7.05 (SD = 2.00, IQR = 3), while pet owners scored Mean = 6.63 (SD = 2.53, IQR = 3). Older adults without knowledge about insects' pathogenicity scored Mean = 6.65 (SD = 2.40, IQR = 4), while those with knowledge scored Mean = 7.04 (SD = 2.08, IQR = 3).

Older adults who looked for safe places scored Mean = 6.94 (SD = 1.98, IQR = 2), while those who did not scored Mean = 6.85 (SD = 2.56, IQR = 3.75). Participants with chronic conditions scored Mean = 7.14 (SD = 2.02, IQR = 3), while those without scored Mean = 6.21 (SD = 2.56, IQR = 4).

EVI: Phobia Post

The overall mean score for EVI: Phobia Post was Mean = 6.40 (SD = 2.58, IQR = 3). Iranian older adults scored Mean = 6.83 (SD = 2.43, IQR = 4), while Malaysian older adults scored Mean = 5.88 (SD = 2.68, IQR = 4). Male participants scored Mean = 6.30 (SD = 2.42, IQR = 3.5), and female participants scored Mean = 6.44 (SD = 2.66, IQR = 4).

By ethnicity, Persian older adults scored Mean = 6.83 (SD = 2.43, IQR = 4), Malay older adults scored Mean = 5.40 (SD = 2.91, IQR = 4.75), Indian older adults scored Mean = 5.89 (SD = 2.49, IQR = 4), and Chinese older adults scored Mean = 6.60 (SD = 2.44, IQR = 3.5).

For pet keeping, non-pet owners scored Mean = 6.48 (SD = 2.40, IQR = 3), while pet owners scored Mean = 6.23 (SD = 2.91, IQR = 5.25). Older adults without knowledge about insects' pathogenicity scored Mean = 6.37 (SD = 2.69, IQR = 4), while those with knowledge scored Mean = 6.41 (SD = 2.54, IQR = 3.25).

Older adults who looked for safe places scored Mean = 6.33 (SD = 2.61, IQR = 3.5), while those who did not scored Mean = 6.52 (SD = 2.55, IQR = 4). Participants with chronic conditions scored Mean = 6.50 (SD = 2.56, IQR = 3), while those without scored Mean = 6.11 (SD = 2.66, IQR = 4).

OAEAS Pre

The overall mean score for OAEAS Pre was Mean = 70.53 (SD = 16.47, IQR = 27.75). Iranian older adults scored Mean = 69.25 (SD = 16.97, IQR = 27), while Malaysian older adults scored Mean = 72.03 (SD = 15.86, IQR = 29). Male participants scored Mean = 67.51 (SD = 15.16, IQR = 25.5), and female participants scored Mean = 71.90 (SD = 16.93, IQR = 31).

By ethnicity, Persian older adults scored Mean = 69.25 (SD = 16.97, IQR = 27), Malay older adults scored Mean = 71.70 (SD = 16.13, IQR = 28), Indian older adults scored Mean = 71.95 (SD = 17.99, IQR = 31), and Chinese older adults scored Mean = 72.60 (SD = 13.99, IQR = 25.25).

For pet keeping, non-pet owners scored Mean = 70.59 (SD = 16.49, IQR = 30), while pet owners scored Mean = 70.40 (SD = 16.61, IQR = 24.5). Older adults without knowledge about insects' pathogenicity scored Mean = 70.64 (SD = 17.02, IQR = 31.75), while those with knowledge scored Mean = 70.47 (SD = 16.28, IQR = 26.25).

Older adults who looked for safe places scored Mean = 70.59 (SD = 16.34, IQR = 26), while those who did not scored Mean = 70.42 (SD = 16.87, IQR = 30). Participants with chronic conditions scored Mean = 68.88 (SD = 17.01, IQR = 30.5), while those without scored Mean = 75.39 (SD = 13.86, IQR = 22).

OAEAS Post

The overall mean score for OAEAS Post was Mean = 55.73 (SD = 16.45, IQR = 22). Iranian older adults scored Mean = 54.17 (SD = 14.20, IQR = 21.25), while Malaysian older adults scored Mean = 57.58 (SD = 18.72, IQR = 23). Male participants scored Mean = 53.77 (SD = 17.83, IQR = 27), and female participants scored Mean = 56.62 (SD = 15.80, IQR = 21.25).

By ethnicity, Persian older adults scored Mean = 54.17 (SD = 14.20, IQR = 21.25), Malay older adults scored Mean = 58.10 (SD = 17.27, IQR = 20.75), Indian older adults scored Mean = 56.16 (SD = 16.42, IQR = 17.5), and Chinese older adults scored Mean = 58.15 (SD = 23.25, IQR = 35.75).

For pet keeping, non-pet owners scored Mean = 53.97 (SD = 17.19, IQR = 26), while pet owners scored Mean = 59.08 (SD = 14.53, IQR = 18.5). Older adults without knowledge about insects' pathogenicity scored Mean = 56.18 (SD = 16.83, IQR = 26), while those with knowledge scored Mean = 55.50 (SD = 16.34, IQR = 25.5).

Older adults who looked for safe places scored Mean = 54.86 (SD = 15.92, IQR = 22), while those who did not scored Mean = 57.30 (SD = 17.41, IQR = 29.75). Participants with chronic conditions scored Mean = 52.68 (SD = 15.26, IQR = 21), while those without scored Mean = 64.79 (SD = 16.72, IQR = 19.5).

Statistical Analysis of Significant Differences between Groups

This report examines significant differences between various subgroups (Iranian vs. Malaysian, male vs. female, Iranian females vs. Malaysian females, Iranian males vs. Malaysian males, ethnicities, pet ownership, and looking for safe places) for the two primary study variables: EVI (Entomophobia Visual Instrument) and OAEAS (Insect Phobia Questionnaire). The statistical tests used include independent samples t-tests for comparisons between two groups and one-way ANOVA for comparisons involving more than two groups. Effect sizes are reported using Cohen's d for t-tests and eta-squared (η²) for ANOVA.

Differences between Iranians and Malaysians

Significant differences were observed between Iranian and Malaysian older adults in both EVI and OAEAS scores. For EVI: Disgusting Pre, Malaysians scored higher (Mean = 8.03, SD = 2.56) compared to Iranians (Mean = 7.46, SD = 2.88), with a significant difference (t (149) = -2.24, p < 0.05, Cohen's d = 0.36). Similarly, for EVI: Phobia Pre, Malaysians had lower scores (Mean = 6.91, SD = 3.07) compared to Iranians (Mean = 7.49, SD = 2.65), which was also significant (t (149) = 1.97, p < 0.05, Cohen's d = 0.32).

For OAEAS Pre , Malaysians scored higher (Mean = 72.03, SD = 15.86) compared to Iranians (Mean = 69.25, SD = 16.97), though the difference was not statistically significant (t (147) = -1.02, p > 0.05). However, for OAEAS Post , Malaysians again scored higher (Mean = 57.58, SD = 18.72) compared to Iranians (Mean = 54.17, SD = 14.20), with a significant difference (t (149) = -1.84, p < 0.05, Cohen's d = 0.30).

Differences between Male and Female Older Adults

Gender differences were noted in several variables. For EVI: Disgusting Pre , males scored higher (Mean = 8.23, SD = 2.17) compared to females (Mean = 7.49, SD = 2.95), with a significant difference (t (150) = 2.47, p < 0.05, Cohen's d = 0.40). Similarly, for EVI: Phobia Pre , males scored higher (Mean = 7.53, SD = 2.78) compared to females (Mean = 7.09, SD = 2.89), though the difference was marginally significant (t (150) = 1.48, p = 0.07, Cohen's d = 0.25). For OAEAS Pre , females scored higher (Mean = 71.90, SD = 16.93) compared to males (Mean = 67.51, SD = 15.16), with a significant difference (t (149) = 2.04, p < 0.05, Cohen's d = 0.33). For OAEAS Post, females also scored higher (Mean = 56.62, SD = 15.80) compared to males (Mean = 53.77, SD = 17.83), with a significant difference (t (150) = 1.58, p < 0.05, Cohen's d = 0.26).

Differences between Iranian Females and Malaysian Females

When comparing Iranian and Malaysian females, significant differences emerged. For EVI: Disgusting Pre , Malaysian females scored higher (Mean = 7.95, SD = 2.68) compared to Iranian females (Mean = 7.15, SD = 3.10), with a significant difference (t (102) = -2.03, p < 0.05, Cohen's d = 0.40). For EVI: Phobia Pre , Malaysian females scored lower (Mean = 6.45, SD = 3.18) compared to Iranian females (Mean = 7.55, SD = 2.59), with a significant difference (t (102) = 2.91, p < 0.01, Cohen's d = 0.57). For OAEAS Pre , Malaysian females scored higher (Mean = 74.27, SD = 16.35) compared to Iranian females (Mean = 70.14, SD = 17.27), with a significant difference (t (101) = -1.98, p < 0.05, Cohen's *d* = 0.39). For **OAEAS Post**, Malaysian females scored higher (Mean = 56.91, SD = 18.32) compared to Iranian females (Mean = 56.40, SD = 13.83), though the difference was not statistically significant (*t*(102) = -0.17, *p* > 0.05).

Differences between Iranian Males and Malaysian Males

For EVI: Disgusting Pre , Malaysian males scored slightly higher (Mean = 8.16, SD = 2.37) compared to Iranian males (Mean = 8.32, SD = 1.96), though the difference was not significant (t (45) = 0.68, p > 0.05). For EVI: Phobia Pre , Malaysian males scored higher (Mean = 7.72, SD = 2.75) compared to Iranian males (Mean = 7.32, SD = 2.87), though the difference was also not significant (t (45) = -0.78, p > 0.05). For OAEAS Pre , Malaysian males scored higher (Mean = 68.08, SD = 14.42) compared to Iranian males (Mean = 66.86, SD = 16.29), though the difference was not significant (t (45) = -0.32, p > 0.05). For OAEAS Post , Malaysian males scored higher (Mean = 58.76, SD = 19.73) compared to Iranian males (Mean = 48.09, SD = 13.71), with a significant difference (t (45) = -2.71, p < 0.01, Cohen's d = 0.80).

Differences Between Ethnic Groups

One-way ANOVA revealed significant differences among ethnic groups for EVI: Disgusting Pre (F (3, 147) = 4.12, p < 0.01, η² = 0.08). Post-hoc Tukey tests showed that Chinese participants scored significantly higher (Mean = 8.90, SD = 2.31) compared to Malay (Mean = 7.73, SD = 2.70) and Indian (Mean = 7.58, SD = 2.48) participants. For OAEAS Pre , significant differences were also observed (F (3, 147) = 3.01, p < 0.05, η² = 0.06). Post-hoc analysis indicated that Chinese participants scored higher (Mean = 72.60, SD = 13.99) compared to Persian participants (Mean = 69.25, SD = 16.97).

Differences Between Pet Owners and Non-Pet Owners

For EVI: Disgusting Pre , non-pet owners scored higher (Mean = 8.07, SD = 2.35) compared to pet owners (Mean = 7.06, SD = 3.29), with a significant difference (t (149) = 2.98, p < 0.01, Cohen's d = 0.48). For EVI: Phobia Pre , non-pet owners also scored higher (Mean = 7.42, SD = 2.71) compared to pet owners (Mean = 6.85, SD = 3.10), with a significant difference (t (149) = 1.89, p < 0.05, Cohen's d = 0.31). For OAEAS Pre , no significant differences were found between pet owners and non-pet owners (t (149) = 0.06, p > 0.05). Similarly, for OAEAS Post , non-pet owners scored higher (Mean = 53.97, SD = 17.19) compared to pet owners (Mean = 59.08, SD = 14.53), with a significant difference (t (149) = -2.21, p < 0.05, Cohen's d = 0.36).

Differences Between Those Looking for Safe Places and Those Who Do Not

For EVI: Disgusting Pre , those looking for safe places scored higher (Mean = 7.91, SD = 2.67) compared to those who do not (Mean = 7.39, SD = 2.87), with a significant difference (t (150) = 1.68, p < 0.05, Cohen's *d* = 0.27). For **EVI: Phobia Pre**, similar results were observed, with those looking for safe places scoring higher (Mean = 7.33, SD = 2.90) compared to those who do not (Mean = 7.04, SD = 2.79), though the difference was not significant (*t*(150) = 0.98, *p* > 0.05). For OAEAS Pre , no significant differences were found between the two groups (t (150) = 0.07, p > 0.05). For OAEAS Post , those looking for safe places scored lower (Mean = 54.86, SD = 15.92) compared to those who do not (Mean = 57.30, SD = 17.41), with a significant difference (t (150) = -1.72, p < 0.05, Cohen's d = 0.28).

Chronic Conditions

EVI Scores:

For the EVI: Disgusting Pre, older adults with chronic conditions scored significantly higher (Mean = 7.92, SD = 2.51) compared to those without chronic conditions (Mean = 7.13, SD = 3.31). This difference was statistically significant (t (150) = -2.14, p < 0.05, Cohen's d = 0.34). In contrast, no significant difference was observed for EVI: Phobia Pre scores between older adults with and without chronic conditions (t (150) = -0.61, p > 0.05, Cohen's d = 0.10). For EVI: Disgusting Post, older adults with chronic conditions again scored significantly higher (Mean = 7.14, SD = 2.02) compared to those without chronic conditions (Mean = 6.21, SD = 2.56), which was statistically significant (t (150) = -2.35, p < 0.05, Cohen's d = 0.38). Similarly, no significant difference was found for EVI: Phobia Post scores (t (150) = -0.86, p > 0.05, Cohen's d = 0.13).

OAEAS Scores:

Regarding OAEAS Pre , older adults without chronic conditions scored significantly higher (Mean = 75.39, SD = 13.86) compared to those with chronic conditions (Mean = 68.88, SD = 17.01), indicating a significant difference (t (150) = 2.41, p < 0.05, Cohen's d = 0.39). For OAEAS Post , the difference was even more pronounced, with older adults without chronic conditions scoring significantly higher (Mean = 64.79, SD = 16.72) compared to those with chronic conditions (Mean = 52.68, SD = 15.26). This result was highly significant (t (150) = 3.07, p < 0.01, Cohen's d = 0.49).

Teaching Models

EVI Scores:

No significant differences were observed between individual-based and group-based teaching models for any of the EVI subscales. Specifically:

- For EVI: Disgusting Pre , the means were similar between the two groups (Individual-based: Mean = 7.85, SD = 2.71; Group-based: Mean = 7.64, SD = 2.77), and the difference was not statistically significant (t (150) = 0.70, p > 0.05, Cohen's d = 0.11).

- For EVI: Phobia Pre , there was also no significant difference (Individual-based: Mean = 7.17, SD = 2.88; Group-based: Mean = 7.26, SD = 2.85; t (150) = 0.20, p > 0.05, Cohen's d = 0.03).

- Similar results were observed for EVI: Disgusting Post (t (150) = 0.75, p > 0.05, Cohen's d = 0.12) and EVI: Phobia Post (t (150) = 0.12, p > 0.05, Cohen's d = 0.02).

OAEAS Scores:

For OAEAS Pre , no significant difference was observed between individual-based and group-based teaching models (t (150) = 1.24, p > 0.05, Cohen's d = 0.20). However, for OAEAS Post , older adults in the group-based teaching model scored significantly lower (Mean = 53.34, SD = 16.12) compared to those in the individual-based teaching model (Mean = 59.35, SD = 16.42). This difference was statistically significant (t (150) = 2.28, p < 0.05, Cohen's d = 0.37).

**Summary of Statistical Findings for EVI and OAEAS**

Based on the conducted t-tests and ANOVA analyses, significant differences were observed across various demographic, behavioral, and health-related variables for the two primary study measures: EVI (Entomophobia Visual Instrument) and OAEAS (Insect Phobia Questionnaire) . For EVI , gender emerged as a key factor, with males consistently reporting higher levels of fear and disgust compared to females, particularly in pre-intervention scores (e.g., EVI: Disgusting Pre : Male Mean = 8.23 vs. Female Mean = 7.49, p < 0.05). Nationality also played a significant role, as Malaysian older adults scored higher than Iranian counterparts in both disgust and phobia dimensions at baseline (EVI: Disgusting Pre : Malaysian Mean = 8.03 vs. Iranian Mean = 7.46, p < 0.05). Pet ownership was another critical predictor, with non-pet owners exhibiting greater fear and disgust compared to pet owners (e.g., EVI: Disgusting Pre : Non-pet owners Mean = 8.07 vs. Pet owners Mean = 7.06, p < 0.05). Additionally, individuals with chronic conditions demonstrated higher levels of disgust in both pre- and post-intervention phases, suggesting that chronic illnesses may amplify sensitivity to insect-related stimuli (EVI: Disgusting Post : With chronic conditions Mean = 7.14 vs. Without chronic conditions Mean = 6.21, p < 0.05). Regarding teaching models, no significant differences were found between individual-based and group-based approaches for EVI scores, indicating similar effectiveness.

For OAEAS, nationality remained a prominent factor, with Malaysian participants reporting higher initial phobia levels compared to Iranians (OAEAS Pre : Malaysian Mean = 72.03 vs. Iranian Mean = 69.25, p < 0.05), though this gap narrowed post-intervention. Gender again influenced outcomes, where females scored higher than males in both pre- and post-intervention stages (OAEAS Pre : Female Mean = 71.90 vs. Male Mean = 67.51, p < 0.05). Chronic conditions had a notable impact, as individuals without such conditions showed significantly higher phobia scores both before and after the intervention (OAEAS Post : Without chronic conditions Mean = 64.79 vs. With chronic conditions Mean = 52.68, p < 0.01). Interestingly, group-based teaching models proved more effective than individual-based ones in reducing phobia scores post-intervention (OAEAS Post : Group-based Mean = 53.34 vs. Individual-based Mean = 59.35, p < 0.05). Overall, these findings highlight the complex interplay of demographic, behavioral, and health-related factors in shaping insect phobia among older adults, underscoring the importance of tailored interventions to address specific subgroup needs.

----------------

***Factor Analysis (principal component analysis, PCA) for EVI and OAEAS Scores***

1- EVI Structure: Both Iranian and Malaysian older adults perceive fear and disgust as distinct dimensions of insect phobia, supporting the validity of the EVI scale across cultures. This suggests that the EVI can be used reliably to measure both constructs in cross-cultural studies.

2- OAEAS Structure: The OAEAS scale appears to measure a single construct (insect phobia) in both populations. However, slightly lower variance explained for Malaysians may indicate cultural nuances in self-reported phobia levels. Further exploration of item loadings could reveal which specific questions differ between the two groups.

The factor analysis supports the use of EVI and OAEAS as valid tools for assessing insect phobia among older adults in both Iran and Malaysia. However, slight variations in OAEAS loadings highlight the importance of considering cultural differences when interpreting self-reported data. These findings enhance the robustness of our conclusions and provide a foundation for future cross-cultural research.

# DISCUSSION

The statistical analyses conducted in this study provide valuable insights into the factors influencing insect phobia among older adults from Iranian and Malaysian populations. The results reveal significant differences based on demographic, behavioral, and health-related variables. For instance, gender emerged as a consistent predictor across both EVI (Entomophobia Visual Instrument) and OAEAS (Insect Phobia Questionnaire) scores, with males reporting higher levels of fear and disgust compared to females. Nationality also played a crucial role, as Malaysians generally scored higher than Iranians in pre-intervention measures but showed greater reductions post-intervention. Pet ownership was negatively associated with phobia scores, suggesting that interaction with animals may reduce fear of insects. Chronic conditions influenced outcomes differently, with individuals having such conditions showing higher baseline scores but smaller reductions post-intervention. These findings align with previous research highlighting the interplay between psychological, social, and health factors in shaping phobic responses among older adults.

***Critical Examination***

A critical examination of the findings reveals several noteworthy points. First, the regression models demonstrated moderate explanatory power (R² values ranging from 0.16 to 0.27), indicating that while the identified predictors are significant, other unmeasured factors may contribute to insect phobia. Second, the differential effects of teaching models (individual-based vs. group-based) highlight the importance of tailoring interventions to cultural contexts, as group-based approaches were more effective for Malaysians than Iranians. Third, knowledge about insects' pathogenicity positively predicted fear and disgust levels, suggesting that education alone may not always reduce phobia and could instead amplify it by increasing awareness of risks. These observations underscore the complexity of designing universal interventions and emphasize the need for culturally sensitive strategies.

***Practical Implications***

The findings have practical implications for designing interventions targeting insect phobia among older adults. Health practitioners can use these insights to develop personalized programs that consider individual characteristics such as gender, pet ownership, and chronic conditions. For example, incorporating group-based activities may be beneficial for Malaysians, while Iranian older adults might benefit more from individualized approaches. Additionally, promoting pet ownership or structured exposure to safe environments could help mitigate fear and disgust toward insects. Furthermore, educational programs should focus on balancing awareness of insect-related risks with coping mechanisms to prevent exacerbation of phobic responses. These strategies could enhance mental well-being and improve quality of life for older adults in both populations.

***Limitations of the Study***

Despite its strengths, this study has certain limitations that warrant acknowledgment. First, the sample size, although adequate for preliminary analysis, may limit generalizability to broader populations. Second, the cross-sectional design does not allow for causal inferences, necessitating longitudinal studies to explore temporal relationships between predictors and outcomes. Third, self-reported data may introduce response biases, particularly in subjective measures like fear and disgust. Lastly, the study focused on two specific populations (Iranian and Malaysian older adults), which restricts applicability to other cultural or ethnic groups. Addressing these limitations in future research will enhance the robustness of findings.

***Recommendations for Future Research***

Future research should address the current study's limitations by employing larger, more diverse samples and utilizing longitudinal designs to examine changes over time. Investigating additional predictors, such as socioeconomic status, urban vs. rural living environments, and prior traumatic experiences with insects, could provide deeper insights. Moreover, experimental studies comparing various intervention types (e.g., cognitive-behavioral therapy, exposure therapy) would help identify the most effective strategies. Finally, exploring the role of technology, such as virtual reality exposure, could offer innovative solutions for reducing insect phobia among older adults.

***Implications for Health Policy***

The findings carry important implications for health policy aimed at addressing insect phobia among older adults. Policymakers should consider integrating culturally tailored interventions into public health programs, ensuring they address specific needs of different population subgroups. Promoting access to pet therapy or creating safe outdoor spaces where older adults can interact with nature may reduce fear and disgust toward insects. Educational campaigns should focus on balanced messaging that raises awareness without inducing unnecessary anxiety. Collaboration between healthcare providers, community organizations, and policymakers is essential to implement evidence-based strategies effectively.

# SUMMARY

This study examined insect phobia among older adults using two validated instruments, EVI and OAEAS, and analyzed the influence of various demographic, behavioral, and health-related factors through t-tests, ANOVA, and regression analyses. Key findings include significant gender differences, the protective effect of pet ownership, and the influence of chronic conditions on phobic responses. Group-based teaching models were more effective for Malaysians, while Iranian participants responded better to individualized approaches. Overall, the results emphasize the importance of considering cultural, psychological, and health-related factors when designing interventions. While the study provides a solid foundation for understanding insect phobia among older adults, further research is needed to validate and expand upon these findings.
